# Self-management action and motivation of Pacific adults in New Zealand with end-stage renal disease

**DOI:** 10.1371/journal.pone.0222642

**Published:** 2019-09-23

**Authors:** Jacqueline Schmidt-Busby, Janine Wiles, Daniel Exeter, Timothy Kenealy

**Affiliations:** 1 Counties Manukau Health, Middlemore Hospital, Auckland, New Zealand; 2 Department of Social and Community Health, School of Population Health, Faculty of Medical and Health Sciences, University of Auckland, Auckland, New Zealand; 3 Department of Epidemiology and Biostatistics, School of Population Health, Faculty of Medical and Health Sciences, University of Auckland, Auckland, New Zealand; 4 School of Medicine, Faculty of Medical and Health Sciences, University of Auckland, Auckland, New Zealand; University of Mississippi Medical Center, UNITED STATES

## Abstract

**Aims:**

To explore actions and motivations for self-management practices of Pacific adults following diagnosis of end stage renal disease (ESRD).

**Methods:**

Focused ethnography using in-depth interviews with 16 Pacific people on haemodialysis for diabetic ESRD, in Auckland, New Zealand. Study participants were of Samoan, Cook Islander, Tongan, Niuean, or Tokelauan ethnicity and aged between 30 to 69 years old. Thematic analysis was used to code and identify emergent themes.

**Results:**

All participants assumed active responsibility for their self-management following their diagnosis of ESRD. They reported positive differences in their current self-management behaviours, compared to pre-ESRD diagnosis. In the face of their terminal diagnosis, participant’s motivations to self-manage their health were fuelled by hope; the hope to live long enough to change their family legacy of diabetes and ESRD. To achieve this, there was a dependency upon family members as a resource for self-management support. Yet at the same time, family members also had health concerns (including diabetes), and several participants themselves were carers for sick or elderly family members.

**Conclusion:**

The growing number of members (within family units) progressing from moderate to late-stage diabetes raises concerns about the sustainability of future family support in Pacific families in New Zealand with histories of diabetes, ESRD, and other chronic diseases. While the burden upon informal carers (family) has been well documented throughout the past few decades, the dynamics of bi-directional carer support between (two or more) sick family members and their families have had less exposure. This has potentially significant implications for Pacific peoples in New Zealand, considering the increases in diabetes prevalence within their families.

## Introduction

Self-management is generally understood as the overall process of an individual’s engagement in their management of one or more chronic diseases [1, 2]. Diabetes and end-stage renal failure (ESRD) requires constant self-management, and care becomes more complex when the patient has co- or multi-morbid conditions. An individual’s behaviour can influence ineffective self-management [3], where behaviour—as an outcome determined by context–shapes how an individual with diabetes acts and copes [4]. Familial influences of how diabetes is understood for example, can facilitate or inhibit the self-management behaviours essential to managing the risk and complications of diabetes [5].

In New Zealand, Pacific peoples have higher rates of diabetes [6] and ESRD [7] compared to other ethnicities, and both diseases are increasingly being seen in multiple successive generations amongst this population. [8] Diabetes is the leading cause of ESRD, with diabetic nephropathy (diabetic kidney disease) the leading cause of ESRD in 72% of Pacific, compared to Māori (68%), Asian (43%), and New Zealand European/Other (36%) [7]. Recent statistics report the incidence of renal replacement therapy (RRT)—including haemo- and peritoneal dialysis, and transplant) in New Zealand is equivalent to 128 per million of population (pmp), a proportion that is similar to Australia (124 pmp) who have a population of approximately 24.6 million. As a group, Pacific RRT rates are significantly higher (471 pmp) compared to New Zealand European (71 pmp), where over the past 5 years RRT for Pacific populations have increased by 14% compared to New Zealand European (3%).[7]

New Zealand’s health care system is a mix of public and private ownership. New Zealand spends 9.5% of its GDP on health compared with an OECD of 8.9%, where public sources account for 80% of overall health spending–well above the OECD average (73%).[9] Secondary and tertiary care—including dialysis—is fully-funded by the government. Primary care is largely provided on a small-business model with substantial government subsidy, but with levels of patient co-payment that limit access to primary care for some patients with diabetes or with renal failure. Medical insurance is optional, and many people use this to pay for private secondary care, but no dialysis is done in private.

In New Zealand, a broad range of diabetes self-management education (DSME) courses and training programmes are offered.[10] Consistent with international recommendations, national guidelines advocate for individuals with diabetes receive structured education that is tailored to meet individual and cultural needs, including personalised guidance on nutrition and physical activity.[11] Pacific DSME non-attendance rates are consistently high.[12, 13] In part, contributing factors include poor cultural fit; many recognised DSME programmes have been developed in populations and setting outside of New Zealand.[14, 15] In a recent study, we found (mis)understandings of disease risk and consequences reinforced the low engagement in diabetes self-management, including attending DSME programmes.[16] We also concluded that health messages delivered by health providers required relevancy to the patients context within which the information is obtained, understood and acted upon. In New Zealand, drug treatments to prevent developing ESRD follows international guidelines[17] to address blood pressure, proteinuria, smoking, and glomulated lipids except that glucose lowering medications for New Zealand patients are limited to metformin, acarbose, sulphonylurea, and insulin.

The most important group in the lives of many Pacific peoples is family, where family refers not only to the immediate or nuclear unit, but also to extended and wider family, where linkages maybe or may not be blood related. Along with Māori, Pacific families in New Zealand are significantly more likely to give support to extended family when compared to other New Zealand families [18]. Through family Pacific individuals are supported socially, mentally, physically, spiritually and culturally [19, 20]. The paradigm from which Pacific families operate is a system of connections, interconnections and relationships where each person plays a role that contributes to the family’s responsibilities, obligations and benefits [21]. Earlier research has reported familial obligations of care remain within the Pacific family system across generations [22], and these values continue today.

In this context, responses to a terminal or life-limiting disease such as ESRD, includes more than the individual’s reaction to the disease. Responses also comprise influences of personal and social contexts [23, 24] such as consideration of family in goal-setting, loss of financial income through cessation of employment, and restrictions within social activities. Personal contexts also influence the motivation to self-manage [25, 26]. Many individuals adjust to living with a life-limiting disease, and hope and motivation have been identified to aid successful adjustment [27–29]. Factors influencing personal responses to self-management do not occur in temporal isolation either; knowledge, beliefs and experiences of life transitions over the life-span for example, can interact to affect motivation and the ability to self-manage [30].

Long standing concerns regarding the health and well-being of Pacific peoples in New Zealand have been raised [31–33]. Factors attributed to influencing their poor health status include a combination of low engagement and participation in healthcare [12, 34, 35]. The findings reported here form part a larger study that sought to understand the lived experiences of Pacific peoples with diabetes and ESRD, so that we might identify opportunities to delay or prevent disease onset and progression. In this manuscript, we report our findings that portray self-management in action following diagnosis of ESRD and describe the motives behind *why* particular self-management practices were undertaken by Pacific adults in New Zealand.

## Methods

### Design, setting, and sampling

This was a qualitative study using focused ethnography. Patient-participants were identified using a purposive maximum variation sampling strategy. The study was undertaken within two in-centre haemodialysis units within a large District Health Board (DHB) in New Zealand, serving a high Pacific Island population. Study inclusion criteria comprised being: of self-identified Samoan, Cook Islander, Tongan, Niuean, or Tokelauan ethnicity; aged 20 years or older who: had type 2 diabetes and ESRD (under-going dialysis); had a good command of the English language; and lived within the DHB catchment area. Study information packs containing a participant information sheet, a copy of the consent form, and a study information flyer were made accessible in general spaces within each unit for prospective patients to read, prior to self-nominating themselves to participate in the study if they so wished.

### Data collection

Field observations, face-to-face interviews, and recorded field notes were undertaken by the first author (JS-B) over a period of 10 weeks in late 2018. All interviews were undertaken (at participants’ requests) during haemodialysis treatments, while participants were either lying on their bed or sitting semi-recumbent in a chair. Field observations—made inside both treatment units, waiting areas, and pick-up/drop-off areas–helped inform analysis and the write-up that follows.

Interviews were undertaken using a relaxed conversational approach that readily accommodated interruptions from nurses who checked monitor readings or attended to monitor alarms. Interviews aimed to explore: understandings of diabetes and kidney disease; health information and communication; self-management; and reflections on diabetes. Although interview data was collected at one point in time from each participant (any time following diagnosis of ESRD), data reflects participants’ opinions, experiences, and reflections across their lives and in relation to their illness (which had changed over time and included symptoms that are experienced as severe). Written informed consent was gained prior to interview and all participants consented to their interview being audio recorded. Interviews were transcribed and all identifying information on transcripts were anonymised prior to being read by those other than the interviewer. Interview transcripts were managed using Nvivo12-Pro and Microsoft Excel.

### Data analysis

Transcripts were read several times by the wider research team. We used a thematic analysis approach informed by Saldaña [36] to go “beyond what is obvious…to explore meaning at a much deeper level” [37], to understand how, why and in what context participant accounts were generated. First and second cycle coding [36] produced initial themes, that were then refined and validated by relating themes back to extracts. Confirmed conceptual themes described participants’ perspectives on self-management and future hopes after being diagnosed with ESRD.

To ensure trustworthiness of the data Lincoln and Guba’s four criteria of credibility, transferability, dependability, and confirmability [38], were integrated into the study design. The authors represent the disciplines of health services (JS-B); health and medical geography (JW; DE), and medicine (TK). JS-B is also Samoan. Advisors additional to the research team represented sociology, diabetes nursing, and Pacific health; they supported specific aspects of coding and interpretation.

### Ethical approval

Institutional ethical approval was obtained from University of Auckland Human Participants Ethics Committee: Reference 020658.

## Findings

Forty-two potential participants showed interest in the study and all were followed up by phone (24) or in person (18). Of those, 9 were ineligible because they did not meet the study criteria, and 11 declined following discussions about the study during the initial meeting. Of the 22 who agreed to take part, six withdrew due to hospitalisation (2) or feeling too unwell (4). A cohort of 16 participants were interviewed, of which half were female and all but three were born in their respective Island nations. The majority (15/16) have been resident in New Zealand for more than 20 years. Participant characteristics are presented in Table 1.

**Table 1:** Participant characteristics.

Code	Age group	Length of illness (estimated years)		Comorbidities
		DM	ESRD	
**Ck.1**	50–59	37	5	DF; CVD
**Ck.2**	40–49	10	1	ED; G; CVD; LD
**Ck.3**	50–59	30	9	ED; CVD; HTN; O(4)
**Ck.4**	50–59	20	1	G; LD; O(1)
**Ck.5**	60–69	38	3	G, LLA; O(2)
**Ck.6**	50–59	13	<1	HTN
**Ni.1**	60–69	27	17	CVD; HTN;
**Sa.1**	40–49*	10	10	A; DF; G; CVD; HTN; LD
**Sa.2**	60–69	32	13	CVD; HTN
**Sa.3**	40–49	19	3	A; ED; HTN; LD; O(1)
**Sa.4**	40–49*	15	5	CVD; HTN,
**Sa.5**	30–39*	9	2	HTN; O(2)
**Tg.1**	30–39	18	2	ED; DF; G; CVD; LLA; O(2)
**Tg.2**	30–39	10	1	ED; HTN
**Tg.3**	60–69	16	3	O(2)
**Tk.1**	50–59	20	3	HTN; O(2)

*New Zealand born

A Asthma; CVD Cardiovascular disease; DF diabetic foot; ED eye disease/cataracts; G Gout; HTN Hypertension; LLA lower limb amputation; LD Liver disease; O Other conditions.

Interviews were between 30- and 80-minutes duration and in English. The shortest interview was stopped after the researcher noticed the participant was becoming increasingly breathless. In total three interviews were re-scheduled due to general unwellness, despite the participant in each case wanting to tell their story. A further three participants requested their interview be conducted during the initial recruitment meeting “*because I might not be here next week*” (Sa.2). All the participants interviewed already had ESRD and were under intensive management. All were considered to be under good metabolic control to the extent possible (from their perspective) considering their existing renal disease and co-morbidities, and were willing to talk with the interviewer about their life and illness(es).

The results are summarised under three themes (1) I can manage my health; (2) Accepting support with self-management; and (3) I hope to live long enough.

### Self-management in action; albeit too late

#### I can manage my health

Before undergoing dialysis, all participants attended ESRD-related education sessions provided by the District Health Board. These sessions were required prior to dialysis as they included information relevant to the dialysis process, such as understanding basic information displayed on the machine’s monitors, navigating the importance of recording weight and blood pressure prior to dialysis, and ‘what you can and cannot do’ when in renal failure. Participants also received individualised education about self-managing their diabetes and other conditions they had. Prior to ESRD they had received more general education and information about diabetes through formalised self-management courses and/or via communication with health providers. Nevertheless, after ESRD-specific education, participants recognised misconceived understandings of diabetes; recounting how insufficient knowledge and understanding about the risks and consequences of the disease influenced poor diabetes self-management and the development of ESRD. Participant also remarked about now knowing symptom causation and management, the functions of different body organs, and how conditions like high blood pressure can affect several organs. Now diagnosed with ESRD, participants realised that self-managing ESRD also comprised self-managing their diabetes and other conditions. This was not an option, but a required activity to prolong life. Most important to many participants was looking after the arm that housed their arteriovenous fistula:

*I can’t do any heavy lifting or anything*. *This is your lifeline—on my arm*. (Sa.5).

*I try to massage it every day–look after it so it doesn’t play up or anything…I try my best to look after it*. *I had one on this arm*, *but I slept on it so that wrecked it*. *Yeah so now I know from that arm I’m not going to do it to this arm* (Tk.1).

An important part of self-managing ESRD included eating the right amount of proteins and limiting intake of fluids and foods containing salt, potassium and phosphates. Prior to ESRD, many participants’ diets were predominantly made up of staples high in these restricted minerals—taro, coconut cream, salty crackers, and salted meats. Moderating the amounts and types of foods challenged taste and eating preferences. Many described attempting to learn how to keep these favourites in their diets:

*Sometimes you crave for the foods that you always have…And you’re not allowed to take more*, *so I try to learn how to do it* (Ck.6).

*I’m not going to lie to you*, *sometimes you know you can’t eat it*, *but to satisfy your eyes and your taste buds*, *yeah I’m going to have a little bit of that* (Sa.5).

While most talked about their trials and errors of getting their regimen right with partners or family members, one participant said they found it helpful to discuss their experiences with others with ESRD who were more experienced with managing food and diet restrictions:

*…I usually had cramps*, *sometimes I’m having problems lying down and can’t breathe*, *and they [friends] told me ‘well*, *if you have too much fluids that’s what happens…* (CK.2).

Several participants said they used to enjoy sports and social activities. However, many physical activities were now no longer available to them. Participants could no longer swim for example in case their fistula or graft became infected. Even simple activities such as enjoying a cup of coffee with friends or enjoying a night out, now needed to be a calculated action because of daily fluid limits:

*When I go nightclub[ing]*, *I don’t drink*. *I always make sure I get me an ice cup and after I’m dancing my mouth gets really dry and then I just have a cube of ice–that’s it* (Sa.3).

*Because at that time*, *I’m fit*. *Ummm*, *I was playing rugby*, *planting*, *or fishing…Now*, *no*. *Because of this [fistula]* (Ck.1).

Dialysis treatments became a ritual for these participants. Scheduled three times per week, many said at times they found attending these treatments physically and mentally stressful—especially when feeling unwell. Several recounted how their general unwellness prior to dialysis was often compounded by feeling poorly with flu and colds during winter months. Others seemed to persevere with symptoms they had no control over:

*I’m always feel so sick before coming to the machine*, *my skin look[s] toxic*, *but I come for my life* (Tg.3).

*I always catch those coughs*, *colds*, *you know the sneezing and dripping nose*, *my head spinning–that on top of feeling sickly is aarggh*.*[gasp]* (Ck.6)

The ability to leave town or go away on holiday required thoughtful planning. For some travelling out of area was not an option. A few, with the support of family, were able to take advantage of ‘holiday’ dialysis units located in Australia; *“I hope and pray the Lord*, *I’ll be 70 next year*, *and book my flight and go to Fiji [for a holiday]”* (Ni.1).

#### Accepting support with self-management

All but four participants lived with partners (and children); three lived with parents or children, and one was *“happy with my own self in my own house”* (Ni.1). Participants expressed how tiring ESRD was, and often felt restricted from fully participating in day-to-day living and dependent on family to support or complete tasks. Many were acutely conscious of how they had become dependent on others for support:

*…when I was younger I moved out of home because I always wanted to have a life*. . . . *But now it’s like even though I say that I want to live on my own*, *which I have no problem with*, *but I think I need someone there*, *in case you know*, *something happens to me* (Sa.1).

*I can do the washing because the washing machine’s doing it*, *and my [partner] takes it out to hang* (Ck.3).

Family members were the main source of support, helping participants face the emotional, physical, and practical challenges of adapting to, and living with ESRD. The one participant who lived alone described following a strict regime to manage ESRD’s side effects and restrictions, “*the routine’s got to be like clockwork*. *Then I know where I am; no more scary*, *no more wondering if I got it right…”* (Ni.1). However like all others, there was acknowledgment of needing family to help.

Several participants living with family, emphasised how their family had relieved the challenges of managing alone. Notwithstanding this, they also felt like a burden:

*I know she’s got two kids–it’s hard for her get up to the other one and come and take me…* (Ni.1).

*…I feel like a burden to my family–my Mum*. *My poor mother*, *she has to do my washing*. *She fusses about me but then she has her own health problems* (Tg.1).

Accepting support with self-management, participants commonly agreed to being “*more focused on me than any other time of my life”* (Sa.5) and this appeared to include an expectation that family particularly, would be there in the future. For one of the oldest participants, this expectation was related to having reached the end of their life as an older adult:

*I’m really OK to go now…I love my family and all the things an old man should be look forward to in old age*, *but I’m ready*. *I’m happy [if] I die in my bed and they [family] will look after it* (Ck.5).

For one of the youngest, family support appeared most important during times of unwellness:

*But now*, *even though I miss out on a few things*, *my life is more important as well as the people that are going to be there when I’m sick* (Sa.5).

Several participants were also faced with accepting that they could no longer work. Dialysis treatments disrupted an ability to work. Although participants reported initially being able to reduce or renegotiate work hours or schedule dialysis during night sessions/non-work hours, the side effects of dialysis (nausea, fatigue, low blood pressure), often meant needing to rest for several hours following. This led to being unable to continue working. For some, the inability to work disrupted their independence, and also meant being unable to financially contribute to the family’s income. This participant described how despite having insufficient income to meet all financial needs, they still tried to keep a healthy diet:

*Only my [partner is] working now*, *so it’s enough to pay our house and some little bit to our fridge*, *and all that sort of thing…we didn’t get it 100% but we get most of things paid*, *but most of the time we run out of money…The can of tuna fish*, *its $3 for a really small can like that*. *Even I have only enough money for three or four cans*, *but we try* (Tg.3).

Friends provided a different kind of support, especially those who also had ESRD. These relationships also offered practical support but allowed participants to vent their frustrations without having to explain the reasons why:

*I started talking with some sort-of friends with it*, *and then just went off*. *I felt really bad that they had to hear it*, *but I felt heaps better inside*. *Needed it*, *that burst*. *I realised after a while that we all did it*, *you know let off*, *and all of us knew exactly how it was* (Tk.1).

#### I hope to live long enough

The news of now having an incurable disease raised mixed emotions among participants: anger, helplessness, grief, confusion, deep regret, and hope. Most told of their disbelief at getting ESRD and were angry at themselves, angry at others, and angry at the world:

*I didn’t like it*. *I felt angry*, *I didn’t want it eh*. *Why would I*? …*But this is the way now* (Ck.3).

*Well when I first found out I thought ‘oh no it will pass*, *I’m sure if I just be good and try the medication again*, *and stick to the insulin it’ll pass*, *it’ll get better*.*’ But they said it doesn’t*, *it doesn’t get better and the damage is done…* (Sa.4).

*It was literally a death sentence* (Ck.4).

What were once future hopes and dreams, were now discarded or repositioned as shorter-term action plans that revolved around daily rituals of dialysis. A small number of participants hoped to secure a kidney transplant in the future. One participant had been waiting thirteen years, “*I’ve been waiting long for a kidney* …*So*, *I just hope to get something soon”* (Sa.2). However, participants hoped to live long enough to fulfil their new plans; hope appeared to be the strongest and most important resource participants used to cope with their current reality: “*I’m too busy to go at the moment…because of my daughter and my family*, *and my hope to help them”* (Tg.1).

Although not discussed specifically, spirituality and the belief in God underpinned most participant interviews. Using gestures and non-verbal communication to complement conversation (a common element of communication within many Pacific cultures[39]), most participants nodded upwards or used upward pointing hand gestures while speaking of hope for the future of family. The few that did explicitly mention God, expressed disappointment in themselves for having made bad decisions about their healthcare. They also viewed the consequences of ESRD (and most likely poor health in general) as not making the most of their life:

*God didn’t give us a brain and a body to waste it* (Ck.5)

*I worry for the younger generations coming through*. *What they will achieve in life being sick…wasting the life that God gave us* (Tg.3)

All participants acknowledged their realisation that members of their family were (or about to) follow(ing) the same path of diabetes and most likely progression to ESRD. With great sadness and regret, several told of how family members had already accepted a life with diabetes. Participants could see the same perceptions and behaviours towards diabetes that lead them to ESRD existed among many of their family members:

*I can see myself in him [sibling]*. *But the thing is he’s killing himself…I’m so sad*. *I hope while I’m alive I can change him*. *I have to*, *everyone else [other family members] thinks they’re going to be alright…* (Sa.3).

*My siblings say they were destined for this too*. *I just shake my head and think ‘what*?*’ I think they’ve just accepted this is how it will be*. *Not while I’m still here*!*… I can accept me being in this situation*, *but not the rest of them*, *no*. (Tk.1).

*We do need to wake-up and realise that what we parents do*, *about health and food and that*, *affects the children for life* (Tg.3).

For those with young families, children gave them the motivation to keep going. Two participants in particular expressed helplessness about dying too soon and found strength in their children to live for as long as possible. Some were adamant that there was no one else but themselves to save their children:

*Some things that go in my mind is like whether I’ll be there for my son…I’m determined to change his life and this family disease or whatever*. *It stops with me*. *It’s made me think about the future–I mean not my one*, *but the future of other families* (Sa.4).

*I’m grateful for that*, *my Mum*. *And my sister*, *cos I know one day she’ll look after my daughter*, *one day far away I hope* (Tg.1).

*At least I can save my baby [daughter]*. *NO*. *I’m not letting this happen to…No this is not happening to my baby*, *that my mission for the rest of my life–my baby* (Ck.4).

The motivation to keep living to benefit others also characterised participants who were caregivers for their ill or elderly family members. In this context (the ‘ill looking after the ill’), caregiving became a bi-directional relationship between participant and family member. Two participants had caregiving roles long-established prior to their ESRD diagnosis, and they explained that this helped ease their situation:

*It’s ok though…[my partner] was first to be sick*. *I think so it’s made it easier for me when I got this*, *to get on with it*, *you know*, *no time to worry about myself*. *Just keep going*. *Who knows what [will] happen next* (Ck.3).

*…but at the moment the old man*, *because he had a minor stroke—so [I’m] trying to look after him and I’m treating my health and it’s just*, *yeah [hard]* (Sa.1).

In contrast, one participant-carer explained how their partner was diagnosed with an aggressive terminal illness shortly after themselves being diagnosed with ESRD. This turned their world upside down, and unlike other ‘bi-directional’ participant-family member ‘carer relationships,’ this caused an uncertainty about practical support in the future:

*Now I’ve got another thing on my plate*, *my partner’s just been diagnosed with terminal cancer*. *So it’s another*, *yeah [thing to cope with] I was stuffed really when [partners name] was diagnosed*, *‘cos that’s my legs at home and now that this has happened*, *[I] don’t know* (Tk.1)

## Discussion

Participants illustrated their emotional responses to the news of having developed an incurable disease; a disease that interrupted their former lives including changes to accommodate regular dialysis treatments. Participants also became actively engaged in self-managing their health after, being diagnosed with ESRD, and receiving health education that was relevant and personal to their needs; positive differences were reported in current self-management behaviours compared to pre-ESRD diagnosis. For participants in the current study, there was a realisation that despite *now* being able to effectively self-manage, their situation of having ESRD may have been preventable. Furthermore, what was now evident to participants, was that family members were repeating behaviours that lead them to ESRD. Motivations for self-managing their health were fuelled by hope; the hope to change future family illness projections, and the hope to live long enough to make this happen. While the participants in this study reported many reactions in line with existing literature, our focus here is on a cluster of responses that constitute new findings.

Much evidence supports the association between good self-management and improved outcomes [4, 23, 40]. For those with ESRD, good self-management is particularly relevant as the incurability of ESRD places disease management (controlling/minimising risk to prolong life) as the primary objective. Attention has been drawn to the difficulties of integrating behavioural and practical changes (needed for managing chronic diseases) into pre-existing life contexts [40–42]. For participants in the current study, the diagnosis of ESRD created a temporal disruption between a past *unhealthy* self and the current *terminal* self. It signalled an approaching end of life and defined the importance of altering their family’s legacy of diabetes; participants did not want future generations to follow in their foot-steps. Previously, misguided attempts at self-management were ever-changing. Misconceived illness representations led to reactive responses to presenting symptom(s) rather than considered management of diabetes. In the present context of a terminal diagnosis, participants assumed responsibility and took purposeful control of their remaining health, rather than being controlled by it.

For many people diagnosed with ESRD, hope is often related to future kidney transplantation [43]. Although some participants expressed their hope for a kidney, all participants hoped to live long enough to educate family members in an attempt to stop diabetes continuing in current and future generations. The outcome of stopping their family legacies of diabetes was contingent upon active self-management; in return, self-managing (ESRD especially), gave them more hope. Duggleby and colleagues contend that hope is a valid process that comprises the future expectations of patients and carers [44]. As a strategy for coping, hope creates an “*uncertain expectation of achieving a future good which*, *to the hoping person*, *is realistically possible and personally significant”* [45].

In contrast, hopelessness, also considered a coping strategy [28], reflects an individual’s estimate that the likelihood of achieving a goal or an outcome is not achievable. As a consequence, it can lead individuals to feeling helpless, disillusioned, despairing, and uncertain [46, 47]. Our participants used hope as an effective coping mechanism to keep from becoming helpless and despairing. This supportive relationship between hope and family is also consistent with Rousseau who suggests, following the initial anguish of being diagnosed with a terminal disease, the meaning attached to (what is left of) life is often defined by family. He further proposes, that an individual’s thoughts and concerns can become less self-centred as *“they envisage*, *and hope for*, *a positive outcome for their family*” [48].

Many participants set *new* future goals such as watching their children grow up or continuing to look after ill or elderly family members. Crucially, time was a controlling mechanism that motivated self-management. Diagnoses of terminal diseases often cause individuals to reframe their meaning of time [49, 50]. Clock or linear time was relevant to participants at the point of diagnosis for context of ESRD severity; but no one could inform participants of exactly how much time they had left. Herth among others describes a refocused time that is defined as future moments, hours, days, months, or non-specific [29, 45]. Mostly, time for participants was socially derived; (re)constructed through living with ESRD and dialysis, and defined by future activities and interactions (experiences) with the environment (dialysis unit, family, self-management) [49, 51].

Participants balanced their uncertain futures with the hope of achieving immediate and future goals. Yet within this constructed temporality, the *uncertainty* could easily become a mechanism that changed hope to hopelessness [52, 53]. For participants waiting to secure a kidney transplant for example, time itself becomes a stressor that could shift hope to increased uncertainty and hopelessness [52, 54]. Waiting creates a sober awareness that while waiting time is running out, simultaneously, time moves slowly while waiting [55]. Only one participant expressed her uncertainty about being alive long enough, to help her young son understand diabetes. Health and social complexities do not necessarily equate to hopelessness [44]. Participants moved beyond their *terminal* self, and found meaning and purpose through reflecting upon what they considered most important to them; their family.

Evidence suggests families re-organise their lives in an attempt to normalise the chronic disease and support the sick family member to continue living a normal life [56]. This was also seen in the current study. However, for study participants and their families, the *re-organisation* was a natural state of everyday family life. Firstly, diabetes was reported by the participants as increasingly prevalent in at least three generations. Therefore, the re-organisation of family life around those with diabetes had become integrated into normative behaviour during the previous two generations. What this meant for current generations, is that rather than in response to symptom exacerbation or illness management [23, 56], the practical, emotional, and financial support offered by families was every-day family culture.

Secondly, although family support prior to ESRD and diabetes was not specifically enquired about in participant interviews, one could also assume, from existing literature [18, 57] and participant narratives, that participants and their families had Pacific (collectivist) value orientations. Interviews revealed many participants lived within multi-generational households, and even the one individual living alone, had regular (if not daily) interactions with family. There was a material and emotional dependence between family members and an obligation of reciprocity of support among family members. During dialysis sessions, the researcher observed that often family members would share support; ‘taking it in turns’ so to speak. Other observations were of practical support in assisting participants with putting on additional clothing or walking to and from the dialysis units; dependencies that would most likely increase in line with disease progression.

### Future implications

The importance of family support has been noted within self-management literature, where for those without family support have increased risk of mortality [58]. The current study findings support the well-established understanding that family supports (carers) work in parallel with formal health services to augment functions of chronic disease management, through for example, self-management support. But what of family members who also have health issues to address and also require support themselves? And what are the consequences for the family? One in three Pacific adults aged 45 years or over now have diabetes and experience more complications than non-Pacific peoples with the condition [59]. In the current study, across generations there were increases in *the ill looking after the ill*. Emotional tensions surrounding the growing number of family members developing late stage diabetes became apparent throughout interviews. Repeat examples of the diagnosis-to-death cycle were recounted by several participants; yet there appeared to be an expectation that family would always be there.

We accept the duration of ESRD varied between respondents and that with time, experiences and self-management may also vary. We acknowledge that full investigation of changing responses over time could best be done within a longitudinal study with repeated interviews and recommend this for future research.

## Conclusions

Participant’s motivations to self-manage their health were fuelled by hope; the hope to stop family members repeating behaviours that lead participants to ESRD, and the hope to live long enough to make this happen. The growing number of members (within family units) progressing from moderate to late-stage diabetes raises concerns about the sustainability of future family support in Pacific families in New Zealand with histories of diabetes, ESRD, and other chronic diseases.

The burden upon informal carers (family) has been well documented throughout the past few decades, where burden has included physical fatigue, emotional exhaustion, depression, anxiety, and financial strain. However, the dynamics of bi-directional carer support between (two or more) sick family members and their families have had less exposure. This has potentially significant implications for Pacific peoples, considering the probable increases in diabetes prevalence within families.

The findings indicate that a partnership with medical professionals will require that they respond to their future patients having considered the (collective) voice of those whose voice has been strongly reported here.

## Supporting information

S1 Table Coding and categorising matrix. (DOCX)
