# Supplementary material for: Self-management action and motivation of Pacific adults in New Zealand with end-stage renal disease
Source: PLoS One. 2019 Sep 23;14(9):e0222642. doi: 10.1371/journal.pone.0222642 (PMC6756531; doi:10.1371/journal.pone.0222642)
Supplement: S1 Table — (DOCX) [file pone.0222642.s001.docx]

**S1 Table. Coding and categorising matrix**

| **FIRST CYCLE CODING (Initial codes)** | **SECOND CYCLE CODING (Categories)** | **THEME** |
| --- | --- | --- |
| Adjusting to ESRD – Emotional  Adjusting to ESRD - Future hopes  Motivations  Supporting family | I hope to live long enough | **Self-management in action – albeit too late** |
| Adjusting to ESRD - Physical  Adjusting to ESRD - Symptoms and understanding  Hiding ESRF from others  Independence  Affordability | I can manage my health |  |
| Dialysis - daily ritual  Self-management: Diet (now)  Self-management: ESRD  Self-management: General (now)  Relationship with Health providers  Family and friend support | Accepting support with self-management |  |
